# Effect of genetic factors on root resorption after orthodontic treatment: A systematic review

**DOI:** 10.6026/9732063002001321

**Published:** 2024-10-31

**Authors:** Hesham Alsaigh, Abdulmajeed Mohammed Al Shujaa, Nuha Hussein Aliuwayed, Rawan Abdullah Alqahtani, Mariya Ibrahim Alibrahim, Areej Mohammad Wadaan, Rema Yousef Alomran

**Affiliations:** 1 Consultant Orthodontist, Dental Department, King Fahad Medical City, Riyadh, Saudi Arabia; 2 Consultant Endodontist, Dental Department, King Fahad Medical City, Riyadh, Saudi Arabia; 3 Intern, College of Medicine and Dentistry, Riyadh Elm University, Riyadh, Kingdom of Saudi Arabia; 4 General Dentist, College of Medicine and Dentistry, Riyadh Elm University, Riyadh, Kingdom of Saudi Arabia

**Keywords:** Root resorption, genetic factors, orthodontic treatment, genetic predisposition

## Abstract

Orthodontic treatment is a widely used dental procedure aimed at correcting mal-alignments for improving oral aesthetics. However, a significant proportion of patient's undergoing orthodontic treatment experience root resorption. This is a complex phenomenon characterized by the loss of tooth root structure. Further, the etiology of root resorption is multifaceted with various factors contributing to its development. Moreover, genetic factors play a crucial role in predisposing individuals to root resorption. Therefore, it is of interest to review the effect of genetic factors on root resorption after orthodontic treatment. The link between certain genetic variants with an increased risk of root resorption in orthodontic patients is of interest to dentists. Known data shows that genetic variants in IL1B, IL-6, and P2RX7 genes contribute to the development of root resorption. Thus, hereditary variables have a substantial impact on the occurrence of root resorption in orthodontic patients. Hence, the need for genetic screening to detect individuals who are susceptible to root resorption is highly relevant. Furthermore, knowledge on genetic risk factors can provide valuable insights for creating customised treatment programmes and preventive measures to reduce the likelihood of root resorption.

## Background:

Orthodontic treatment, a widely employed dental procedure aimed at correcting malalignments and improving oral aesthetics, has become an integral component of modern dentistry, with an estimated 1 in 5 individuals requiring orthodontic intervention globally [1, 2-3]. While orthodontic treatment has revolutionized the field of dentistry, enabling the correction of various malocclusions and improving oral function, it is not without its complications, with root resorption being a significant concern [4- 5]. Root resorption, a complex and multifaceted phenomenon characterized by the loss of tooth root structure, can lead to compromised tooth integrity, tooth loss, and diminished oral function, ultimately impacting the quality of life of affected individuals. The etiology of root resorption is intricate, with various factors contributing to its development, including genetic predisposition, mechanical stress, hormonal influences, and other environmental factors [6]. Genetic factors, in particular, are thought to play a crucial role in predisposing individuals to root resorption, with certain genetic variants conferring an increased risk of root resorption [7]. The identification of genetic susceptibility loci and the elucidation of the underlying molecular mechanisms are essential for the development of personalized treatment plans and preventative strategies to minimize the risk of root resorption [8- 9]. EARR is a multifactorial phenomenon that results from the intricate interaction of host and environmental factors. The genetic aspect of EARR has attracted a lot of interest, and the laboratory mouse is a useful model organism for studying the genetic foundation of EARR. High levels of homology between the mouse genome and human gene sequences, along with synteny-the preservation of gene order-make it easier to identify orthologous genes [10]. The mouse is a desirable model for researching the genetics of EARR because it has a dense genetic map and can be modified by transgenic and gene targeting techniques [11, 12].

Orthodontically induced EARR is a significant concern in orthodontic practice, with approximately 30% of patients exhibiting significant EARR and 2-5% experiencing severe root resorption [13, 14]. The outcome of tooth movement during orthodontics is influenced by the balance between applied pressures and the adaptive reaction of the alveolar bone. Biomechanical risk factors, including tooth extraction, appliance type, and treatment length, contribute to the variance in root resorption [15]. The local release of inflammatory mediators, particularly those involved in the interleukin-1 path way, and the disruption of osteogenic and osteolytic cell function are thought to influence alveolar bone remodeling and modeling [16].

Numerous association studies have investigated the relationship between genetic variations and EARR, with a heritability estimate of 50-84% based on twin and sib-pair studies [17]. While no single genetic variation has been consistently associated with EARR, certain loci, including those involved in the interleukin-1 pathway, bone cell differentiation, and mechanotransduction pathways, have been implicated in EARR susceptibility [17]. For example, genetic variations in the IL-1 receptor antagonist, IL-1RN, and the purinergic receptor P2RX7 genes have been associated with EARR [17-18]. Therefore, it is of interest to determine the effect of genetic factors on root resorption among patients undergoing orthodontic treatment.

## Materials and Methods:

## Eligibility criteria:

A comprehensive literature review was conducted from 1997 to 2023 utilising the PubMed, Medline and Science Direct databases. The utilised keywords were "genetic factors", "root resorption", and "orthodontic treatment". The PRISMA (version 2019) flowchart was utilised to depict the process of selecting the articles that were searched (Figure 1 - see PDF). The study proposal for this review has been evaluation by the Institutional Review Board (IRB) of Riyadh Elm University (REU) and has been allocated the registration number "FUGRP/2023/333/1032". Table 1 shows the different selection criteria utilized for the review.

## Search strategy:

The search strategy was designed to capture all pertinent studies published in the English language between 1997 and 2023. Three electronic databases, namely Science Direct Library, Web of Science and PubMed, were systematically searched to identify studies that met the pre-specified inclusion criteria. The search strings employed in each database were crafted using a combination of Boolean operators and MeSH keywords to ensure a thorough and targeted search. The resulting selection process schematics are elucidated through (Table 1).

## Data extraction process:

The extracted data was organized into three primary categories: study attributes, participant attributes, and outcome metrics. Study attributes encompassed information on the research design, publication date, country of origin, and language of publication. Participant attributes included data on the sample size, age range, sex distribution, and treatment characteristics related to orthodontic care (such as type and duration of treatment). The outcome metrics category was further divided into two subcategories: genetic components and root resorption outcomes. Genetic components included data on the specific genes and variants examined, the methods used for genetic testing, and the results of the genetic analyses (including odds ratios and confidence intervals). Root resorption outcomes included data on the definition and measurement of root resorption, the prevalence and severity of root resorption, and the correlation between genetic components and root resorption outcomes.

## Bias assessment protocol:

Each study included in this review had its risk of bias assessed using the Cochrane Risk of Bias Assessment Tool, as Table 2 illustrates.

## Results:

Analyses of the included studies [6, 19, 20, 21, 22, 23, 24, 25, 26-27] reveal the connection between several genes and the likelihood of root resorption in orthodontic patients, as shown in Table 3 and Table 4 respectively.

## Genetic factors and EARR:

Harris *et al.* [6] found that genetic factors play a significant role in EARR, with heritability estimates averaging 70% for three roots. Baghaei *et al.* [21] identified specific genetic variations, such as the G.G. genotype of IL-1A rs1800587 that increased the risk of EARR in certain patients. Iglesias *et al.* [22] found a significant correlation between homozygous IL1B gene subjects and an increased risk of post-orthodontic EARR. Ciurla *et al.* [23] demonstrated that specific haplotypes in P2RX7 and IL1RN significantly altered the risk of EARR development. Linhartova *et al.* [24] found that a specific haplotype in P2RX7 modified the risk of EARR in children.

## Treatment-related factors and EARR:

Sharab *et al.* [19] found a significant association between prolonged treatment duration and EARR. Guo *et al.* [20] found that a combination of factors, including sex and root movement, can increase the risk of EARR.

## Vitamin D receptor gene polymorphism and EARR:

Fontana *et al.* [25] investigated the link between TaqI vitamin D receptor gene polymorphism and EARR, finding a higher incidence of EARR in patients with certain genotypes.

## Multiple factors and EARR:

Iglesias-Linares *et al.* [26] found that homozygous subjects for a specific polymorphism in the osteopontin gene cluster were protected against post-orthodontic EARR. Marañón *et al.* [27] examined the relationship between seven SNPs across four genes and EARR, finding potential associations between genetic factors and EARR.

## Discussion:

This study's unique aspect is the analysis of multiple genetic factors involved in the IL1B expression pathway, along with treatment-related variables, to better understand their combined impact on EARR incidence during standard orthodontic treatment using a specific appliance. The role of age and gender in EARR development remains unclear, as previous research has not provided conclusive evidence [28-29]. We examined the findings of previous studies and identified high-risk factors for EARR, including sex, root mobility, genetic variations, and others. Previous clinical investigations used two-dimensional measurement techniques, such as X-ray films and panoramic radiographs, to assess root resorption. However, EARR is a three-dimensional phenomenon that can occur anywhere along the root. Our analysis revealed that the average EARR was approximately 12.59 cubic millimetres, and nearly 35% of patients exhibited resorption greater than 15 cubic millimetres. These results suggest that EARR is a common and significant condition, which may explain why orthodontists are increasingly concerned about it [30]. The intricate interaction between hereditary and environmental factors influencing the development of EARR during orthodontic treatment has been clarified by recent investigations. Malocclusions, which are defined by irregular teeth alignments and placements, appear to be more the result of acquired developmental conditions than of hereditary features, despite previous notions to the contrary [31]. This discovery has significant implications for understanding the relationship between EARR and malocclusion severity. To further elucidate this relationship, a comprehensive analysis of 11 dento-skeletal characteristics was conducted using pre-treatment cephalograms. The results indicated that the type of malocclusion has a negligible impact on the estimation of EARR, suggesting that genetic regulation of EARR operates independently of malocclusion type [32]. Previous studies have highlighted racial and ethnic disparities in EARR susceptibility, with Caucasians and Hispanics exhibiting higher rates of root resorption compared to Asians. Our sample comprised a diverse population, with 74% Caucasians, 16% African Americans, 6% Hispanics, 3% Asians, and 2% South Asians. Although the sample size was insufficient to establish a statistically significant correlation between EARR and racial/ethnic groups, a trend emerged indicating higher rates of root resorption in Caucasians, Hispanics, and South Asians compared to African Americans and Asians [32]. Demographic analysis revealed that age and sex were not reliable predictors of EARR development during orthodontic treatment, consistent with previous findings [32].

Furthermore, our investigation explored the association between genetic polymorphisms and EARR susceptibility. A notable correlation was observed between the P2RX7 (rs208294) polymorphism and apical resorptions, with a higher frequency of CC polymorphism in patients with apical resorptions compared to the control group [33, 34- 35]. A statistical investigation revealed a significant correlation between the Lavender and Malmgren's index and the P2RX7 (C/T) polymorphism, with advanced grades of resorption more frequent in patients with CC and C.T. genotypes compared to T.T. genotypes [36-37]. Our research emphasises how crucial it is to take into account the interactions between EARR pathophysiology, orthodontic tooth movement, and genetic variants. Orthodontic tooth movement is known to change the expression of genes that respond to vitamin D in periodontal ligament cells; this effect is known to be time-dependent during therapy [38-40]. Our analyses support the hypothesis that inherited alleles of the interleukin one cytokine agonist and antagonist genes may contribute to genetic susceptibility to EARR in this ethnic group and validate the correlation between IL1B polymorphisms and EARR susceptibility in orthodontic patients from different regions [41, 42- 43]. The development of targeted medicines and advancements in orthodontic therapy for high-risk patients may be facilitated by the identification of biomarkers that predict EARR risk.

Comparing our results to those of other studies conducted in this area [39, 43, 44, 45- 46], a number of parallels and differences become apparent. Both our study and the studies of Yu *et al.* [45] and Liu *et al.* [44] consistently showed the importance of genetic variables in the development of EARR following orthodontic treatment. Furthermore, the genes linked to EARR in our investigation, including IL1B, IL-6, P2RX7, and others, were also connected to the investigations conducted by Yu *et al.* [45] and Liu *et al.* [44]. Furthermore, the results of Kalra *et al.* [39] and Liu *et al.* [44] corroborated the role of inflammatory and immune response pathways in EARR, which is what our study indicated. In terms of study design, Liu *et al.* [44] utilised an extreme phenotypic analysis to find genetic variants linked to severe EARR, whereas our work used a case-control strategy to find genetic connections with EARR. Using a different strategy, Yu *et al.* [45] looked at the genetic variables affecting the length of orthodontic treatment. In the meanwhile, Silva and colleagues [46] carried out a more thorough investigation, examining the interplay between several factors, such as therapy and clinical characteristics, as well as genetic variations in the susceptibility to OIEARR. The intricate interaction between genetic and environmental variables in EARR was better understood because to the latter strategy. Our research, along with that of Liu *et al.* [44] and Yu *et al.* [45], concentrated more intently on the genetic aspects of EARR. Using a more theoretical approach, Kalra *et al.* [39] gave a narrative assessment of the biochemical pathways connected to EARR and the roles different genes play in its development.

## Limitations:

The limitations of this study are multifaceted and warrant consideration when interpreting the findings. Firstly, the study's reliance on existing literature may have introduced potential biases and limitations inherent to the original studies. The heterogeneity of the included studies, in terms of study design, population characteristics, and outcome measures, may have compromised the precision and generalizability of the findings. Furthermore, our focus on genetic factors may have overlooked the complex interplay between genetic and environmental factors contributing to root resorption. The lack of standardized genotyping and phenotyping protocols across studies may have introduced inconsistencies in the data, potentially affecting the accuracy of the results. Additionally, the study's scope was limited to a specific set of genes, which may not exhaustively capture the genetic landscape of root resorption.

## Clinical recommendations:

In light of the limitations and the observed findings, several recommendations can be derived to advance our understanding of the genetic factors contributing to root resorption. Firstly, future studies should strive to adopt standardized genotyping and phenotyping protocols to ensure consistency and comparability across studies. Secondly, the scope of genetic investigation should be broadened to encompass a more comprehensive range of genes and genetic variants. Thirdly, studies should aim to recruit diverse populations to enhance the generalizability of the findings. Fourthly, the complex interplay between genetic and environmental factors should be explored to provide a more nuanced understanding of root resorption. Finally, efforts should be made to develop and validate biomarkers that can accurately predict an individual's risk of developing root resorption, enabling personalized orthodontic treatment strategies.

## Conclusion:

Known data helps to understand the impact of specific genetic polymorphisms on root resorption among orthodontic patients. Genetic polymorphisms such as rs208294, IL1B, IL-6 SNP rs1800796, rs530537, IL1RN, P2RX7, rs731236, rs9138 and rs11730582 are known to be associated with root resorption among orthodontic patients.

## Figures and Tables

**Table 1:** Inclusion and exclusion criteria devised for the review

**Study characteristics**	**Inclusion criteria**	**Exclusion criteria**
Research design	Case-control studies, Randomized control trials	Systematic reviews, Meta-analyses, Expert opinions, Narrative reviews, Survey-based investigations
Language of publication	English	Non-English languages
Investigation type	*In vivo* human studies	In vitro investigations, Animal studies, Laboratory experiments
Publication date	Published between 1997 and 2023	Published before 1997, Published after 2023

**Table 2:** Summary of Cochrane Risk of Bias Assessment

**Study**	**Selection Bias/Appropriate control selection/baseline characteristics similarity**	**Selection bias in randomization**	**Selection bias in allocation concealment**	**Performance-related bias in blinding**	**Reporting bias/Selective reporting of outcomes**	**Detection bias Blinding outcome assessors**	**Accounting for confounding bias**
Sharab *et al.* [19]	+	+	+	-	+	+	+
Guo *et al.* [20]	-	+	+	+	+	+	+
Harris *et al.* [6]	+	+	-	+	+	+	+
Baghaei *et al.* [21]	+	+	+	+	+	+	+
Iglesias *et al.* [2]	+	+	-	+	+	+	-
Ciurla *et al.* [23]	+	-	+	+	+	+	+
Linhartova *et al.* [24]	+	+	+	+	+	+	+
Fontana *et al.* [25]	+	+	+	+	+	-	+
Iglesias-Linares *et al.* [26]	+	+	+	+	+	+	+
Marañón-Vásquez *et al.* [27]	+	-	+	+	+	+	+

**Table 3:** Sample characteristics of the selected studies

**Author (year)**	**Population**	**Sample (N)**	**Mean age**	**Orthodontic technique**	**Methods used to detect root resorption**	**Imaging exams**	**Evaluated teeth**	**Angle malocclusion**
Sharab *et al.* [19]	Caucasian	460	15.78 > 1.13	Fixed edgewise appliances	Malmgren's grading system	Pre- and post-treatment lateral cephalometric, panoramic, and occlusal radiographs	Four permanent maxillary incisors	Class II
Guo *et al.* [20]	Chinese	174	14.0> 7	Straight archwire technique	Mimics software	Panoramic, lateral cephalometric, CBCT	Maxillary incisors.	Class I Class II Class III
Harris *et al.* [6]	Caucasian	103	14.1	standard edgewise technique	Linge and Linge, McFadden	Pre- and post-treatment panoramic	maxillary central incisors, mandibular central incisors, and left and right mandibular first molars	Class I Class II Class III
Baghaei *et al.* [21]	Caucasian	195	16.2 y	Fixed edgewise appliances	Dolphin Imaging software	cephalometric radiograph	Maxillary incisors.	Class I Class II Class III
Iglesias *et al.* [22]	Caucasian	44	22 years⁄ 9 months	Straight archwire technique	Linge and Linge's method	pre- and post-treatment lateral cephalometric and panoramic radiographs	Maxillary incisors.	Not mentioned
Ciurla *et al.* [23]	Caucasian	101	21.08 years (± 7.32 years).	Fixed edgewise appliances	Malmgren and Levander scores	pre- and post-treatment lateral cephalometric and panoramic radiographs	Maxillary incisors.	Class II
Linhartova *et al.* [24]	Caucasian	99	15.2 > 5.3 years)	Not mentioned	Linge and Linge's method	orthopantomograms and lateral cephalometric radiographs	Maxillary incisors.	Not mentioned
Fontana *et al.* [25]	Caucasian	377	Group 2: 14.50 Group 2: 15.33 Group 3: 16.46	edgewise or straight-wire techniques	Electronic digital vernier caliper; Utustools Professional	periapical x-rays	Maxillary incisors.	Class II div I
Iglesias-Linares *et al.* [26]	Caucasian	87	24.70 5.95	straight-wire technique	Linge and Linge's method	lateral cephalometric and panoramic radiographs	Not mentioned	Class I Class II Class III
Marañón-Vásquez *et al.* [27]	Caucasian	143	13.5 ± 4.5	straight-wire technique	Image J software	panoramic and/or cephalometric radiographs	Upper central incisors, lower first molars	Class I Class II Class III

**Table 4:** Description of the selected studies-genetic polymorphisms evaluated

**Author (year)**	**Genes and genetic polymorphisms evaluated**	**Genetic polymorphism in association with root resorption**
Sharab *et al.* [19]	P2RX7, rs208294, rs1718119, rs2230912, rs580253, CASP1/ICE, IL1b, IL1RA, rs419598	P2RX7, rs208294 P2RX7, rs1718119 P2RX7, rs2230912 CASP1/ICE, rs580253 IL1RA, rs419598
Guo *et al.* [20]	IL-6 SNP, rs1800796 GC, IL-1RN SNP, rs419598	IL-6 SNP, rs1800796 GC IL-1RN SNP, rs419598
Harris *et al.* [6]	type I SSQ, type III SSQ	Not mentioned
Baghaei *et al.* [21]	IL-1A, IL-1B, IL-1RN, P2RX7, CASP1, CASP1	IL-1A, IL-1B IL-1RN, P2RX7 IL-1A, CASP1 IL-1B, CASP1
Iglesias *et al.* [22]	IL1-b, rsll43634, IL1-a, rsl800587, IL1-RN, rs419598	IL1-b +3953 (rsll43634) L1-a -889 (rsl800587) IL1-RN+2018 (rs419598)
Ciurla *et al.* [23]	P2RX7, IL1RN	P2RX7 (C/T) IL1RN (C/T)
Linhartova *et al.* [24]	IL-17A, P2RX7, rs2275913, P2RX7, rs1718119, Thr348Ala, TNFRSF11B, rs2073618	IL-17A -197G/A P2RX7 +489C/T P2RX7 +1068G/A TNFRSF11B -163T/C
Fontana *et al.* [25]	T.T., T.C., CC	TaqI polymorphism (rs731236)
Iglesias-Linares *et al.* [26]	rs9138, rs11730582, 4q21-q25	CC vs CA/AA AA vs CA/CC CA vs CC/AA
Marañón-Vásquez *et al.* [27]	rs7975232, rs731236, rs1544410, rs7975232rs1544410, rs7975232, rs731236	rs7975232-rs731236 rs1544410-rs7975232 rs1544410-rs7975232-rs731236
